# Long non-coding RNA CASC2 restrains high glucose-induced proliferation, inflammation and fibrosis in human glomerular mesangial cells through mediating miR-135a-5p/TIMP3 axis and JNK signaling

**DOI:** 10.1186/s13098-021-00709-5

**Published:** 2021-08-26

**Authors:** Dongju Zhu, Xiang Wu, Qian Xue

**Affiliations:** 1 Department of Nephrology, The Affiliated Hospital, Panzhihua University, Panzhihua, 617000 Sichuan China; 2 Department of Pediatrics, Panzhihua Central Hospital, Panzhihua, 617000 Sichuan China; 3 Department of Gastroenterology, The First Affiliated Hospital of Chongqing Medical University, Chongqing, 400000 China

**Keywords:** Diabetic nephropathy, High glucose, CASC2, miR-135a-5p, TIMP3

## Abstract

**Background:**

Diabetic nephropathy (DN) is a common complication of diabetes. Long non-coding RNA (lncRNA) cancer susceptibility candidate 2 (CASC2) is reported to exert a protective role in DN by a previous study. The working mechanism underlying the protective role of CASC2 in DN progression was further explored in this study.

**Methods:**

The expression of CASC2 and microRNA-135a-5p (miR-135a-5p) was determined by real-time quantitative polymerase chain reaction (RT-qPCR). Cell proliferation ability was assessed by Cell Counting Kit-8 (CCK8) assay and 5-ethynyl-29-deoxyuridine (EDU) assay. Enzyme-linked immunosorbent assay (ELISA) was conducted to analyze the production of inflammatory cytokines in the supernatant. Western blot assay was performed to analyze protein expression. Dual-luciferase reporter assay and RNA immunoprecipitation (RIP) assay were performed to verify the target relationship between miR-135a-5p and CASC2 or tissue inhibitors of metalloproteinase 3 (TIMP3).

**Results:**

High glucose (HG) treatment reduced the expression of CASC2 in human glomerular mesangial cells (HMCs) in a time-dependent manner. CASC2 overexpression suppressed HG-induced proliferation, inflammation and fibrosis in HMCs. miR-135a-5p was validated as a target of CASC2, and CASC2 restrained HG-induced influences in HMCs partly by down-regulating miR-135a-5p. miR-135a-5p bound to the 3ʹ untranslated region (3ʹUTR) of TIMP3, and CASC2 positively regulated TIMP3 expression by sponging miR-135a-5p in HMCs. miR-135a-5p silencing inhibited HG-induced effects in HMCs partly by up-regulating its target TIMP3. CASC2 overexpression suppressed HG-induced activation of Jun N-terminal Kinase (JNK) signaling partly through mediating miR-135a-5p/TIMP3 signaling.

**Conclusions:**

In conclusion, CASC2 alleviated proliferation, inflammation and fibrosis in DN cell model by sponging miR-135a-5p to induce TIMP3 expression.

**Supplementary Information:**

The online version contains supplementary material available at 10.1186/s13098-021-00709-5.

## Background

Diabetes is a metabolic disease featured by high level of blood sugar over a prolonged period [1]. Diabetic nephropathy (DN) is a serious complication of diabetes, and it is an important inducing factor of cardiovascular disorders and end-stage renal failure [2]. Hyper-proliferation of mesangial cells and deposition of extracellular matrix (ECM) contribute to DN progression [3, 4]. Understanding the molecular mechanism behind the aberrant pathological features of mesangial cells is essential to identify novel targets for DN treatment.

Previous study found that long noncoding RNAs (lncRNAs) are widely dysregulated in DN [5]. lncRNAs have been identified as novel targets in the diagnosis, prognosis, and therapy of DN [6]. Accumulating studies reported that lncRNAs regulate the phenotypes of mesangial cells by sponging microRNAs (miRNAs). For instance, Li et al. found that KCNQ1OT1 silencing alleviates high glucose (HG)-mediated proliferation, oxidative stress, and deposition of ECM in mesangial cells through mediating miR-18b/HMGA2 signaling [7]. Wang et al. demonstrated that lncRNA CTBP1-AS2 attenuates HG-mediated oxidative stress, the deposition of ECM, and inflammatory response in mesangial cells by sponging miR-155-5p to induce FOXO1 expression [8]. lncRNA cancer susceptibility candidate 2 (CASC2) is reported to suppress DN progression through different signaling axes [9, 10]. In this study, the working mechanism of CASC2 in DN progression was further explored.

miRNAs are implicated in the regulation of cell biological behaviors by binding to the 3ʹ untranslated region (3ʹUTR) of target messenger RNAs (mRNAs), causing the translational repression or degradation of mRNAs [11]. Accumulating evidence have uncovered the regulatory roles of miRNAs in the development of diabetes and its associated complications, including DN [12, 13]. Through bioinformatics prediction, miR-135a-5p was a potential target of CASC2. miR-135a-5p expression is reported to be markedly up-regulated in DN patients relative to that in control group [14]. Zhang et al. demonstrated that miR-135a-5p expression is enhanced in the serum and renal tissue samples of DN patients, and miR-135a-5p absence alleviates transforming growth factor β1 (TGF-β1)-mediated renal fibrosis in DN [15]. In this study, we tested the target relationship between CASC2 and miR-135a-5p and investigated their functional correlation in DN progression.

Tissue inhibitors of metalloproteinases (TIMPs) are identified as endogenous specific inhibitors of matrix metalloproteinases (MMPs) [16]. The conversion of ECM is modulated by the dynamic balance between the biological activities of MMPs and TIMPs [17]. TIMP3 was predicted as a potential target of miR-135a-5p by bioinformatics database. TIMP3 is the most highly expressed TIMP in kidney, and it is implicated in the regulation of cell inflammatory response and fibrosis [18]. It is reported that TIMP3 expression is reduced in mice with diabetes, and TIMP3 silencing contributes to DN development [19, 20]. Here, the interaction between miR-135a-5p and TIMP3 was tested, and their functional correlation in DN progression was explored.

HG-induced human glomerular mesangial cells (HMCs) was used as in vitro DN experimental model as previously reported [21–23]. We explored the biological function of lncRNA CASC2 and its associated mechanism in DN progression using DN cell model.

## Materials and methods

### Cell lines

Human glomerular mesangial cells (HMCs) and 293T cells were acquired from BeNa Culture Collection (Beijing, China). Cells were cultured in Dulbecco’s modified Eagle’s medium (DMEM) medium (Gibco, Carlsbad, CA, USA) plus 10% fetal bovine serum (FBS, Gibco) and 1% penicillin-streptomycin (Sigma, St. Louis, MO, USA) at 37 °C with 5% CO_2_. HMCs exposed to high glucose (HG; final concentration of 25 mM; Sigma) or normal glucose (NG; final concentration of 5.5 mM) for 48 h were utilized as DN cell model.

### Real-time quantitative polymerase chain reaction (RT-qPCR)

RNA samples were isolated with Trizol reagent (Invitrogen, Carlsbad, CA, USA). To measure the level of miR-135a-5p, reverse transcription was implemented using TaqMan microRNA Reverse Transcription kit (Invitrogen), and RT-qPCR was implemented using the specific primers (Table 1) and SYBR mix reagent (Takara, Dalian, China). Complementary DNA (cDNA) of CASC2 was synthesized using High-Capacity cDNA Reverse Transcription kit (Invitrogen) followed by thermal cycle reaction with commercial SYBR mix reagent (Takara). Glyceraldehyde-3-phosphate dehydrogenase (GAPDH) acted as the house-keeping gene for CASC2, whereas U6 acted as the house-keeping gene for miR-135a-5p. The fold changes of gene expression were analyzed by the 2^−∆∆Ct^ method.

**Table 1:** Specific primers for RT-qPCR

Gene	Primer sequences (5ʹ-3ʹ)
CASC2	Forward primer: AGCCAGAAAATGCATGACACA
	Reverse primer: TGGCTCCTCACATCTCCAGT
miR-135a-5p	Forward primer: GCCGAGTATGGCTTTTTATT
	Reverse primer: GCAGGGTCCGAGGTATTC
U6	Forward primer: GCTTCGGCAGCACATATACTAAAAT
	Reverse primer: CGCTTCACGAATTTGCGTGTCAT
GAPDH	Forward primer: TATGATGACATCAAGAAGGTGGT
	Reverse primer: TGTAGCCAAATTCGTTGTCATAC

### Cell transfection

The overexpression plasmid of CASC2 (oe-lncRNA CASC2) was constructed by our laboratory, and empty pcDNA 3.1 (+) vector (vector; Invitrogen) was utilized as the control. The pcDNA 3.1 (+) vector was digested by Kpn I and Xho I, and the longest transcript of CASC2 (NCBI Reference Sequence: NR_026939.1) was amplified by qPCR and inserted into the vector. miR-135a-5p mimic (5ʹ-UAUGGCUUUUUAUUCCUAUGUG-3ʹ), mimic negative control (mimic NC; 5ʹ-UAGGGCUAGCGUGACGUAAUUG-3ʹ), miR-135a-5p inhibitor (5ʹ-GUCACACAUGUGACCGUGUUA-3ʹ), inhibitor NC (5ʹ-GUUUUGUGCACCAGUGAACA-3ʹ), small interfering RNA (siRNA) against TIMP3, including si-TIMP3#1 (5ʹ-UCCUUUACCAGCUUCUUCCCC-3ʹ), si-TIMP3#2 (5ʹ-AUCUUCAUCUGCUUGAUGGUG-3ʹ), and si-TIMP3#3 (5ʹ-ACAUCUUGCCAUCAUAGACGC-3ʹ) along with si-NC (5ʹ-UGAUAGACCCGUAAGAUGCAA-3ʹ) were purchased from GenePharma (Shanghai, China). When cell confluence reached about 60%, transfection was performed with Lipofectamine 2000 (Invitrogen).

### Cell Counting Kit-8 (CCK8) assay

The optical density was measured to generate proliferation curve to analyze the proliferation capacity of HMCs. HMCs in 96-well plates in the indicated time points were incubated with CCK8 reagent (Dojindo, Tokyo, Japan) for 2 h, and the absorbance (450 nm) was read using the microplate reader (Invitrogen).

### 5-ethynyl-29-deoxyuridine (EDU) assay

HMCs in 24-well plates were incubated with EDU reagent (Sigma) for 2 h. After washing with phosphate buffered saline (PBS) solution (Sangon Biotech, Shanghai, China) twice, cells were immobilized with 4% paraformaldehyde (EpiZyme, Shanghai, China) and incubated with 0.5% Triton X-100 (EpiZyme). DAPI dye reagent (EpiZyme) was added to each well to stain the nucleus. Five random fields at the magnification of 200× were selected. The numbers of EDU^+^ cells (proliferative cells) and DAPI^+^ cells (total cells) were analyzed, and the ratio of positive cells was calculated as EDU^+^ cells/DAPI^+^ cells.

### Enzyme-linked immunosorbent assay (ELISA)

The concentrations of monocyte chemotactic protein 1 (Mcp-1), tumor necrosis factor α (TNF-α), and interleukin 6 (IL-6) in the supernatant were assessed using their matching kits (R&D Systems, Minneapolis, MN, USA).

### Western blot assay

Total protein samples (30 µg) from HMCs were loaded onto the 10% sodium dodecyl sulfate-polyacrylamide gel electrophoresis (SDS-PAGE), and blotted on polyvinylidene difluoride (PVDF) membrane (Millipore, Billerica, MA, USA). PBS tween-20 (PBST; Sangon Biotech) solution containing 5% skimmed milk was utilized to block the non-specific sites of the membrane. After that, the membrane was labeled with diluted primary antibodies of proliferating cell nuclear antigen (PCNA; ab18197; 1:8000; Abcam, Cambridge, MA, USA), CyclinD1 (ab16663; 1:10,000; Abcam), TGF-β1 (T3176; 1:8000; Sigma), fibronectin (FN; ab2413; 1:5000; Abcam), collagen 4 (Col-4; C1926; 1:5000; Sigma), TIMP3 (ab276134; 1:5000; Abcam), Jun N-terminal Kinase (JNK; ab110724; 1:8000; Abcam), phosphorylated JNK (p-JNK; T183; 1:3000; ab47337) and GAPDH (ab9485; 1:20,000; Abcam). After labeling with horseradish-peroxidase (HRP)-conjugated secondary antibody (1:5000; Abcam), protein bands were visualized using several films and the Super Signal West Pico Chemiluminescent Substrate Kit (Pierce, Rockford, IL, USA). Quantification of protein bands was performed using the Image Lab analysis software (Bio-Rad, Hercules, CA, USA), and the intensities of protein bands were normalized to GAPDH.

### Establishment of lncRNA/miRNA/mRNA axis

LncBase database (http://carolina.imis.athena-innovation.gr/diana_tools/web/index.php?r=lncbasev2/index-predicted) was utilized to predict the possible miRNA targets of CASC2, and the possible mRNA targets of miR-135a-5p were predicted by StarBase database (http://starbase.sysu.edu.cn).

### Dual-luciferase reporter assay

The target interaction between miR-135a-5p and CASC2 or TIMP3 was verified by dual-luciferase reporter assay. The partial fragment of CASC2 or TIMP3, including the predicted miR-135a-5p binding sites or matching mutant binding sites, was inserted into the downstream of pmirGLO vector (Promega, Madison, WI, USA). The re-constructed luciferase plasmids were termed as lncRNA CASC2 wt, lncRNA CASC2 mut, TIMP3 3ʹUTR wt and TIMP3 3ʹUTR mut. These luciferase plasmids were co-introduced with miR-135a-5p mimic or its control into 293T cells. After transfection for 48 h, Firefly luciferase activity and Renilla luciferase activity were determined using the Dual-Luciferase reporter assay system kit (Promega). Renilla luciferase activity was utilized as the control.

### RNA immunoprecipitation (RIP) assay

RIP assay was conducted to extract target-RNA complexes using the Magna RIP RNA-binding Protein Immunoprecipitation kit (Millipore). HMCs were disrupted using the RIP buffer. The antibody against Argonaute 2 (Ago2; Millipore) or immunoglobulin G (IgG; Millipore) was incubated with protein A/G beads for 1 h at 4 °C. The antibody-pre-coated beads were incubated with cell lysates, and the levels of CASC2 and miR-135a-5p were examined by RT-qPCR.

### Statistical analysis

Data analysis was performed using GraphPad Prism 7.0 software (GraphPad, La Jolla, CA, USA). Normally distributed data were expressed as mean ± standard deviation (SD). The differences between groups were analyzed by unpaired Student’s *t*-test (in two groups) or one-way analysis of variance (ANOVA) followed by Tukey’s post hoc test (in multiple groups). *P < *0.05 was designated as statistically significant.

## Results

### HG treatment promotes the proliferation, inflammation, and fibrosis of HMCs partly by reducing the level of CASC2

**Fig. 1:**
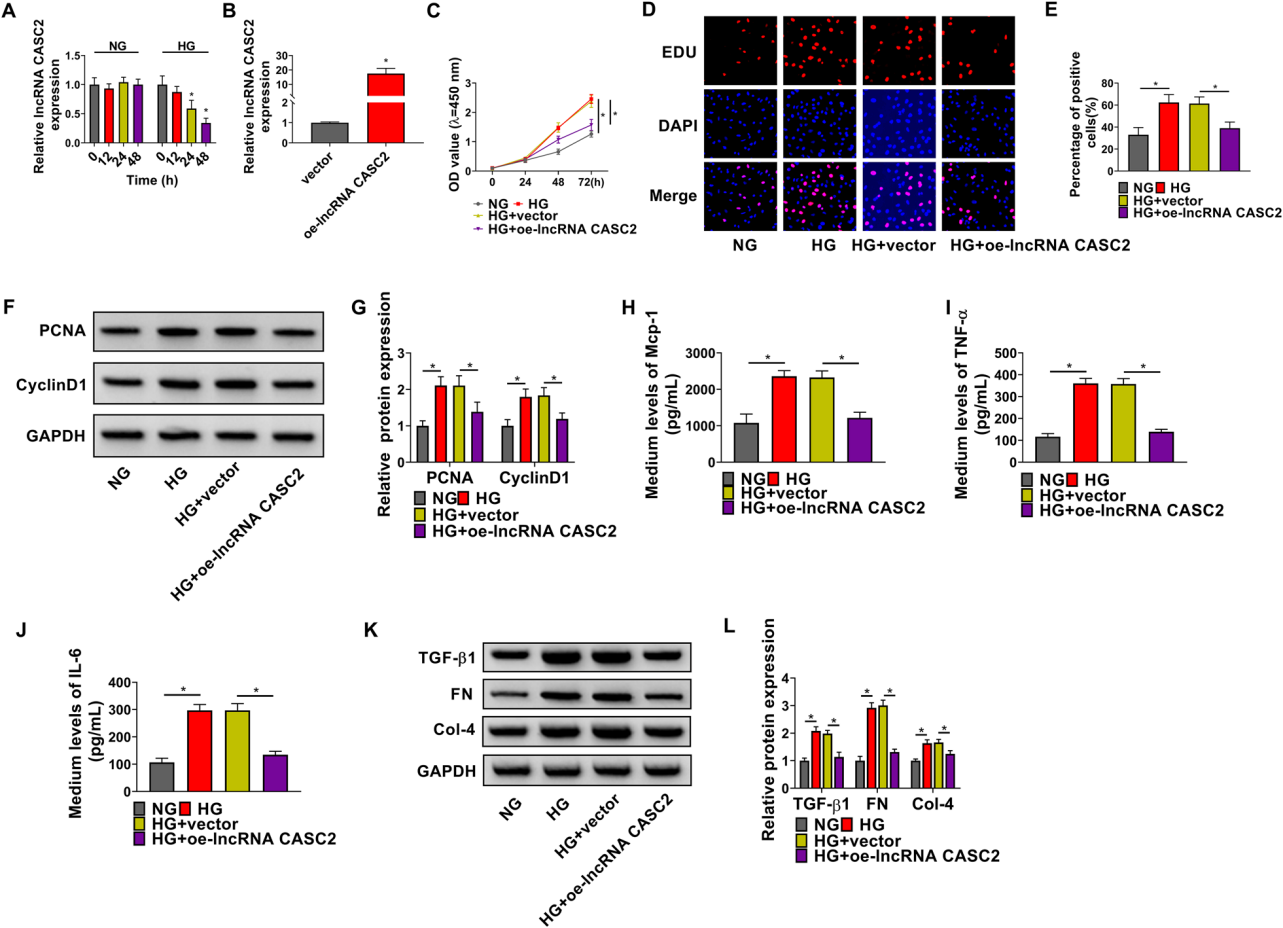
HG treatment promotes the proliferation, inflammation, and fibrosis of HMCs partly by reducing the level of CASC2. **A** The expression of CASC2 was determined in HMCs stimulated by NG (5.5 mM) or HG (25 mM) for 0 h, 12 h, 24 or 48 h by RT-qPCR. **B** RT-qPCR was applied to verify the overexpression efficiency of CASC2 plasmid (oe-lncRNA CASC2) in HMCs. **C**–**L** HMCs were transfected with vector or oe-lncRNA CASC2 followed by HG treatment. **C** CCK8 assay was applied to analyze cell proliferation ability. **D**, **E** EDU assay was performed to assess the proliferation ability of treated HMCs. **F**, **G** Western blot assay was applied to measure the expression of proliferation markers (PCNA and CyclinD1) in HMCs. **H–J** ELISA was conducted to evaluate the release of Mcp-1, TNF-α and IL-6 in the medium of HMCs. **K**, **L** The levels of TGF-β1, FN and Col-4 in HMCs were determined by Western blot assay. *P < 0.05

HG (25 mM) treatment decreased the level of CASC2 in HMCs in a time-dependent manner, while NG (5.5 mM) treatment had no significant effect on CASC2 expression in HMCs (Fig. 1A). To test whether the down-regulation of CASC2 was important for HG-induced effects, we rescued the expression of CASC2 using its overexpression plasmid (oe-lncRNA CASC2) in HG-treated HMCs. The overexpression efficiency of oe-lncRNA CASC2 was high in HMCs (Fig. 1B). CCK8 assay displayed that HG treatment promoted the proliferation of HMCs, which was partly attenuated by the overexpression of CASC2 (Fig. 1C). EDU assay presented that HG exposure promoted the proliferation of HMCs, evidenced by the increased ratio of EDU^+^ cells (Fig. 1D, E). Moreover, HG-induced promoting effect on the proliferation of HMCs was attenuated by the addition of CASC2 plasmid (Fig. 1D, E). HG exposure up-regulated the expression of proliferation-associated proteins (PCNA and CyclinD1), and the overexpression of CASC2 reduced the levels of PCNA and CyclinD1 (Fig. 1F, G). Cell inflammatory response was assessed by measuring the release of inflammation-associated cytokines (Mcp-1, TNF-α and IL-6) via ELISA. HG treatment induced the release of Mcp-1, TNF-α and IL-6, and cell inflammatory response was partly attenuated by CASC2 overexpression in HMCs (Fig. 1H–J). Three fibrosis-associated proteins (TGF-β1, FN and Col-4) were detected by Western blot assay to analyze the fibrosis. HG treatment up-regulated the expression of fibrosis-associated proteins (TGF-β1, FN and Col-4), which was partly alleviated by the overexpression of CASC2 in HMCs (Fig. 1K, L). These data suggested that HG-induced pro-proliferative, pro-inflammatory and pro-fibrotic effects were partly dependent on the down-regulation of CASC2.

### miR-135a-5p is a target of CASC2

**Fig. 2:**
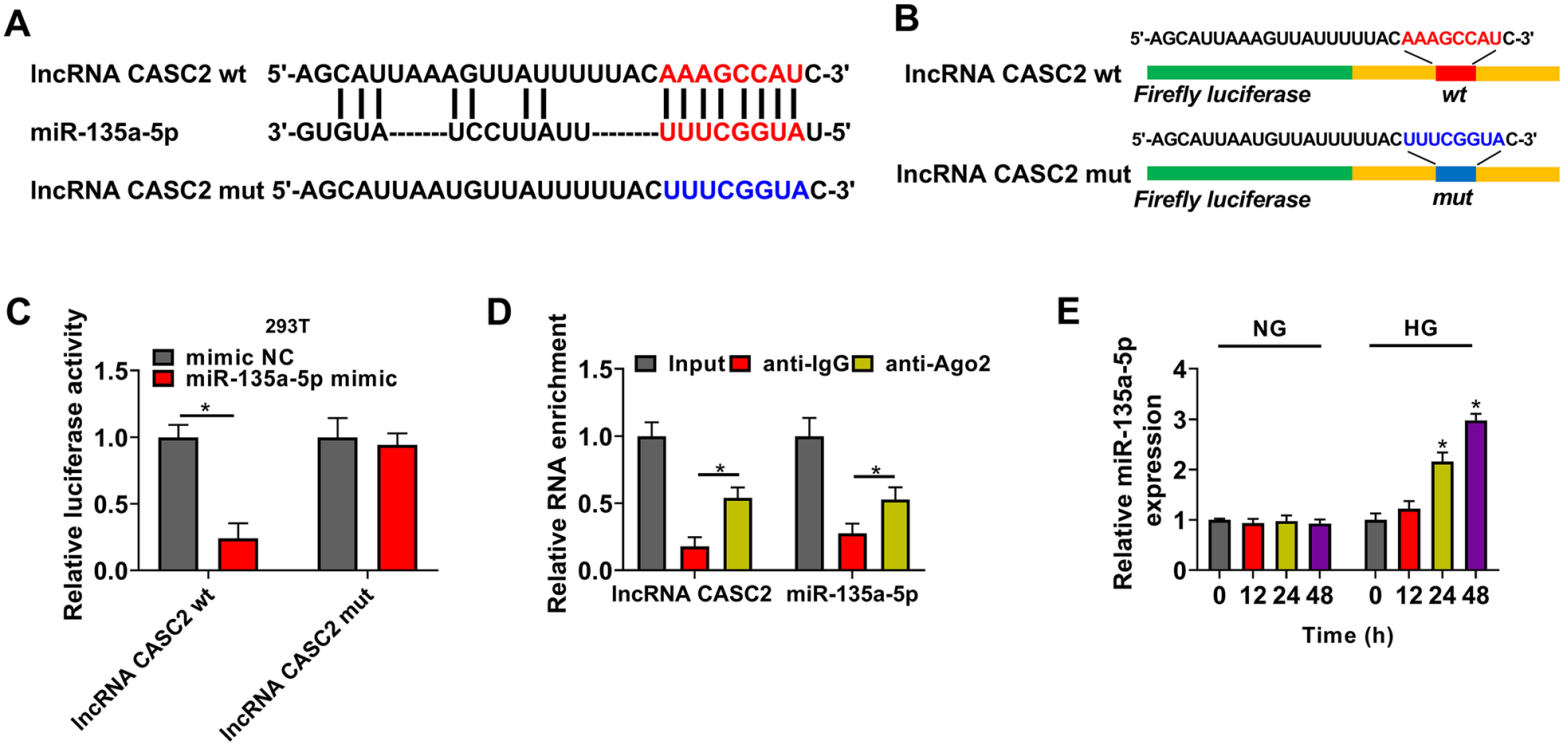
miR-135a-5p is a target of CASC2. **A** Bioinformatics database LncBase was utilized to predict CASC2-miRNA interactions, and miR-135a-5p was predicted to be a potential target of CASC2. The complementary sites between miR-135a-5p and CASC2 were shown. **B** The partial fragment of CASC2, containing the wild-type (wt) or the mutant type (mut) putative binding sites with miR-135a-5p, was amplified and cloned downstream of Firefly luciferase gene in luciferase vector to generate lncRNA CASC2 wt or lncRNA CASC2 mut. **C** 293T cells were co-transfected with luciferase plasmids and miR-135a-5p mimic or mimic NC, and luciferase activities in four groups were determined to test the interaction between miR-135a-5p and CASC2. **D** RIP assay was carried out to test the binding relation between miR-135a-5p and CASC2 in HMCs. **E** The level of miR-135a-5p was examined in HMCs induced by NG or HG for 0 h, 12 h, 24 or 48 h by RT-qPCR. *P < 0.05

We predicted the potential miRNA targets of CASC2 using LncBase database, and miR-135a-5p was a candidate target of CASC2 on the basis of their complementary sites (Fig. 2A). We cloned the fragment of CASC2, containing the wild-type or the mutant type binding sites with miR-135a-5p, into the downstream of Firefly luciferase gene to obtain luciferase reporter plasmid lncRNA CASC2 wt or lncRNA CASC2 mut (Fig. 2B). The luciferase activity of wild-type plasmid (lncRNA CASC2 wt) was markedly reduced by the overexpression of miR-135a-5p, while the luciferase activity of mutant plasmid (lncRNA CASC2 mut) was unchanged by the addition of miR-135a-5p or mimic NC (Fig. 2C), suggesting the interaction between CASC2 and miR-135a-5p in 293T cells. RIP assay was conducted to verify the target relationship between CASC2 and miR-135a-5p in HMCs. When using Ago2 antibody, both CASC2 and miR-135a-5p were enriched (Fig. 2D), demonstrating the target interaction between CASC2 and miR-135a-5p in RNA-induced silencing complex (RISC). HG treatment time-dependently increased the expression of miR-135a-5p in HMCs (Fig. 2E). These results revealed that miR-135a-5p was a target of CASC2 in HMCs.

### CASC2 overexpression restrains the proliferation, inflammation and fibrosis of HG-induced HMCs partly by down-regulating its target miR-135a-5p

**Fig. 3:**
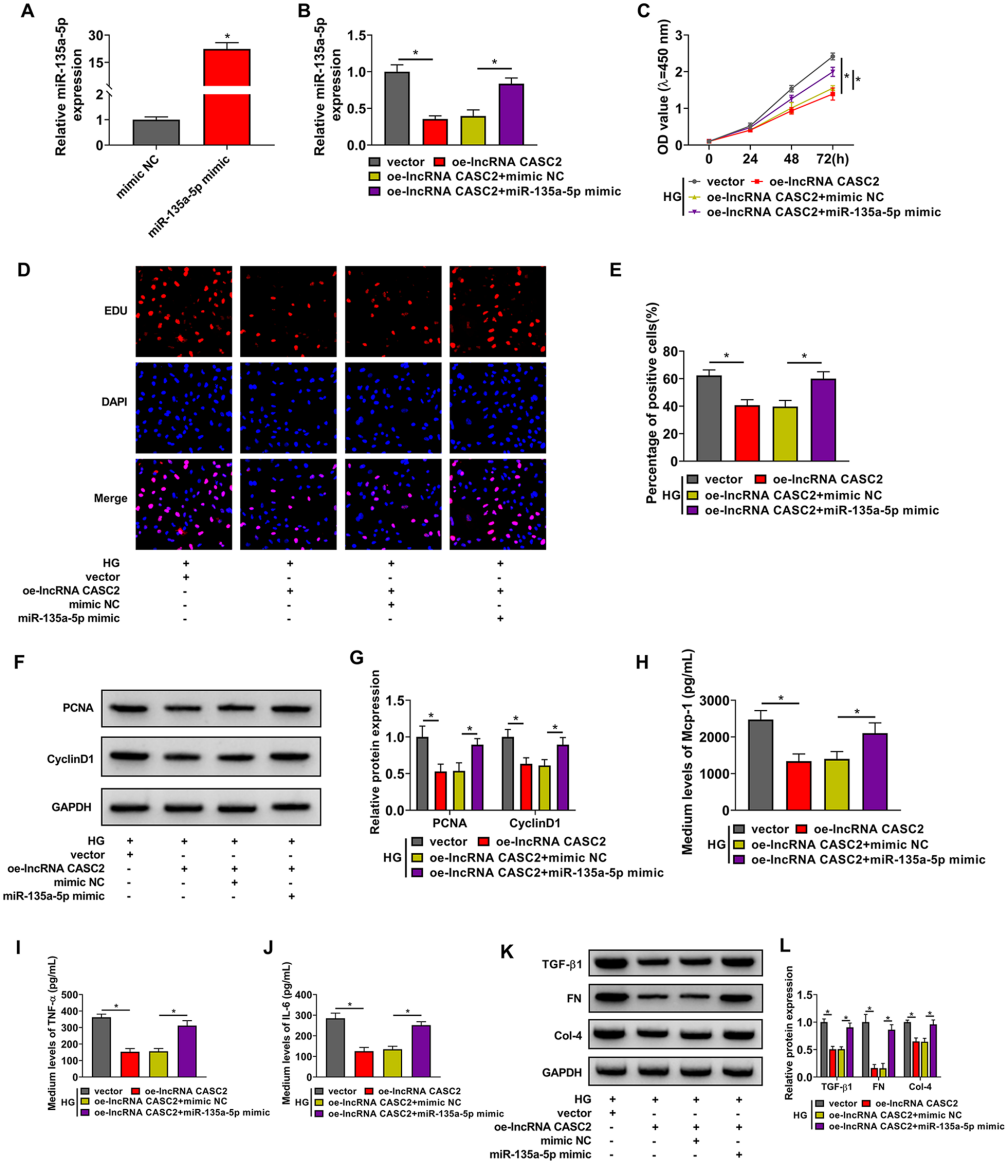
CASC2 overexpression restrains the proliferation, inflammation and fibrosis of HG-induced HMCs partly by down-regulating its target miR-135a-5p. **A** The expression of miR-135a-5p was determined in HMCs transfected with miR-135a-5p mimic or mimic NC by RT-qPCR. **B** HMCs were transfected with oe-lncRNA CASC2 alone or together with miR-135a-5p mimic. The expression of miR-135a-5p was examined by RT-qPCR. **C**–**L** HMCs transfected with oe-lncRNA CASC2 alone or together with miR-135a-5p mimic were induced by HG. **C** Cell proliferation was analyzed by CCK8 assay. **D**, **E** EDU assay was implemented to analyze cell proliferation ability. **F**, **G** The levels of proliferation-associated markers (PCNA and CyclinD1) were determined by Western blot assay. **H**–**J** The production of inflammation-associated cytokines was analyzed by ELISA. **K**, **L** Western blot assay was applied to measure the expression of extracellular matrix (ECM) proteins, including TGF-β1, FN and Col-4. *P < 0.05

The overexpression efficiency of miR-135a-5p mimic was high in HMCs (Fig. 3A). CASC2 overexpression reduced the level of miR-135a-5p (Fig. 3B), suggesting the negative regulatory relationship between CASC2 and miR-135a-5p in HMCs. To explore whether CASC2 exerted its function by targeting miR-135a-5p, we performed rescue experiments. The addition of miR-135a-5p mimic largely rescued the level of miR-135a-5p in CASC2-overexpressed HMCs (Fig. 3B). Through CCK8 assay and EDU assay, we found that CASC2 overexpression-mediated suppressive effect on cell proliferation was largely counteracted by the addition of miR-135a-5p mimic (Fig. 3C–E). CASC2 overexpression-mediated suppressive effects on the levels of pro-proliferation proteins (PCNA and CyclinD1) were largely counteracted by the overexpression of miR-135a-5p (Fig. 3F, G). miR-135a-5p overexpression largely rescued the production of inflammatory cytokines in CASC2-overexpressed HMCs upon HG treatment (Fig. 3H–J). The expression of fibrosis-associated proteins (TGF-β1, FN and Col-4) was down-regulated in CASC2-overexpressed HMCs upon HG treatment, and the accumulation of miR-135a-5p largely rescued the levels of TGF-β1, FN and Col-4 (Fig. 3K, L). These data suggested that CASC2 overexpression suppressed HG-induced proliferation, inflammation and fibrosis of HMCs partly by down-regulating miR-135a-5p.

### miR-135a-5p binds to the 3ʹUTR of TIMP3

**Fig. 4:**
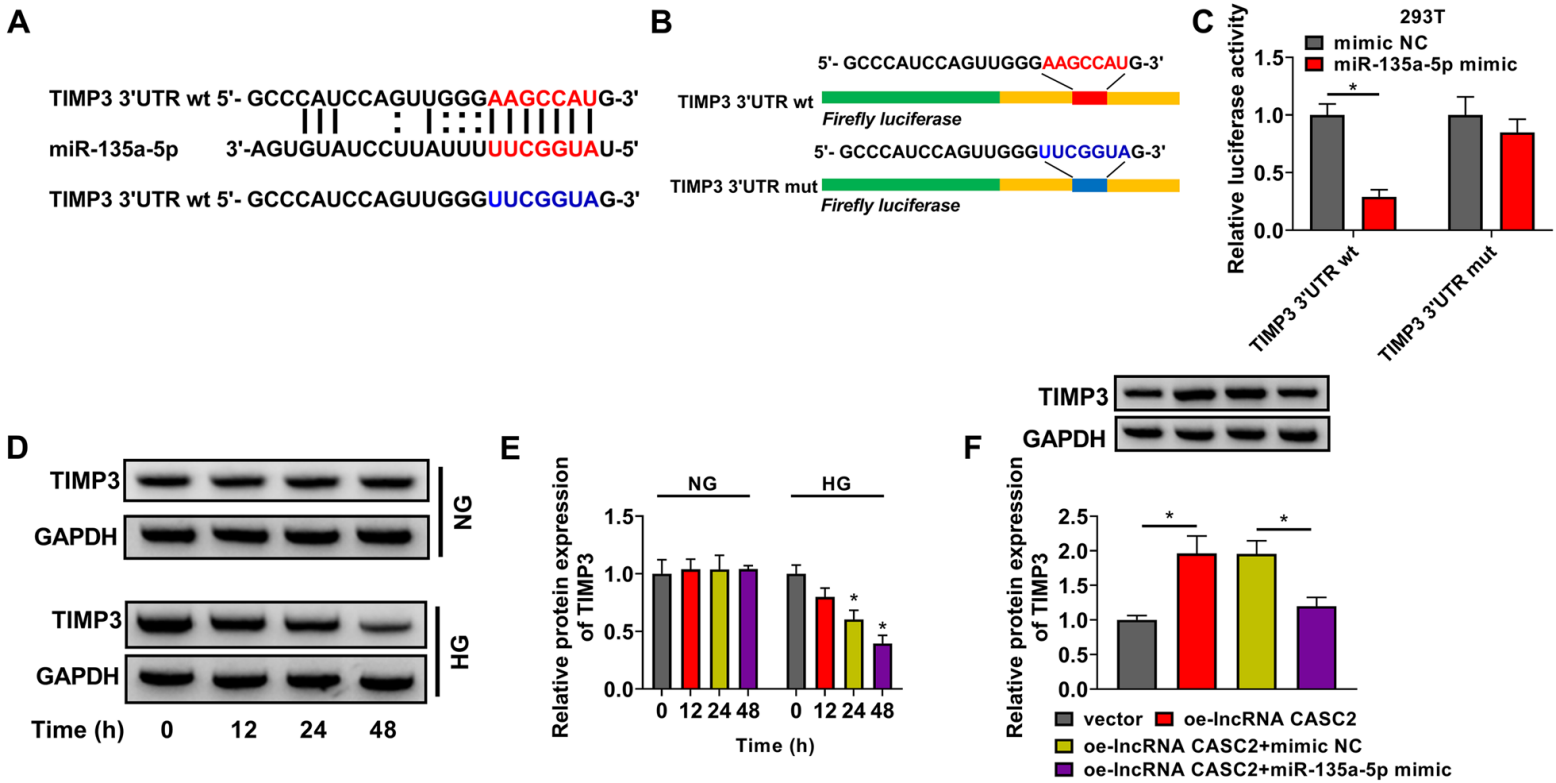
miR-135a-5p binds to the 3ʹUTR of TIMP3. **A** The potential interaction between miR-135a-5p and TIMP3 was predicted by StarBase database. **B** The 3ʹUTR fragment of TIMP3, containing the wt or mut putative binding sites with miR-135a-5p, was inserted downstream of the Firefly luciferase gene in luciferase vector to obtain TIMP3 3ʹUTR wt and TIMP3 3ʹUTR mut. **C** The target relation between miR-135a-5p and TIMP3 in 293T cells was tested by dual-luciferase reporter assay. **D**, **E** The protein level of TIMP3 was determined in HMCs induced by NG or HG for 0 h, 12 h, 24 or 48 h by Western blot assay. **F** The protein level of TIMP3 was measured in HMCs transfected with vector, oe-lncRNA CASC2, oe-lncRNA CASC2 + mimic NC or oe-lncRNA CASC2 + miR-135a-5p mimic by Western blot assay. *P < 0.05

The mRNA targets of miR-135a-5p were predicted by StarBase database, and the putative binding sites between miR-135a-5p and TIMP3 were shown in Fig. 4A. We constructed luciferase reporter plasmid named TIMP3 3ʹUTR wt or TIMP3 3ʹUTR mut that contained the wild-type or the mutant type predicted binding sites with miR-135a-5p (Fig. 4B). Luciferase activity was dramatically reduced in 293T cells in TIMP3 3ʹUTR wt group when co-transfected with miR-135a-5p mimic rather than mimic NC (Fig. 4C), suggesting the target interaction between miR-135a-5p and TIMP3. When the putative binding sites in TIMP3 were mutated, the luciferase activity was no longer reduced by the overexpression of miR-135a-5p (Fig. 4C), suggesting that miR-135a-5p bound to TIMP3 via the putative sites. HG treatment decreased the protein level of TIMP3 in a time-dependent manner in HMCs (Fig. 4D, E). CASC2 overexpression up-regulated the protein expression of TIMP3, which was partly attenuated by the accumulation of miR-135a-5p in HMCs (Fig. 4F), suggesting that CASC2 up-regulated TIMP3 expression partly by sponging miR-135a-5p. Overall, TIMP3 was confirmed as a target of miR-135a-5p, and it was regulated by CASC2/miR-135a-5p signaling.

### miR-135a-5p knockdown suppresses HG-induced effects in HMCs partly by up-regulating TIMP3

**Fig. 5:**
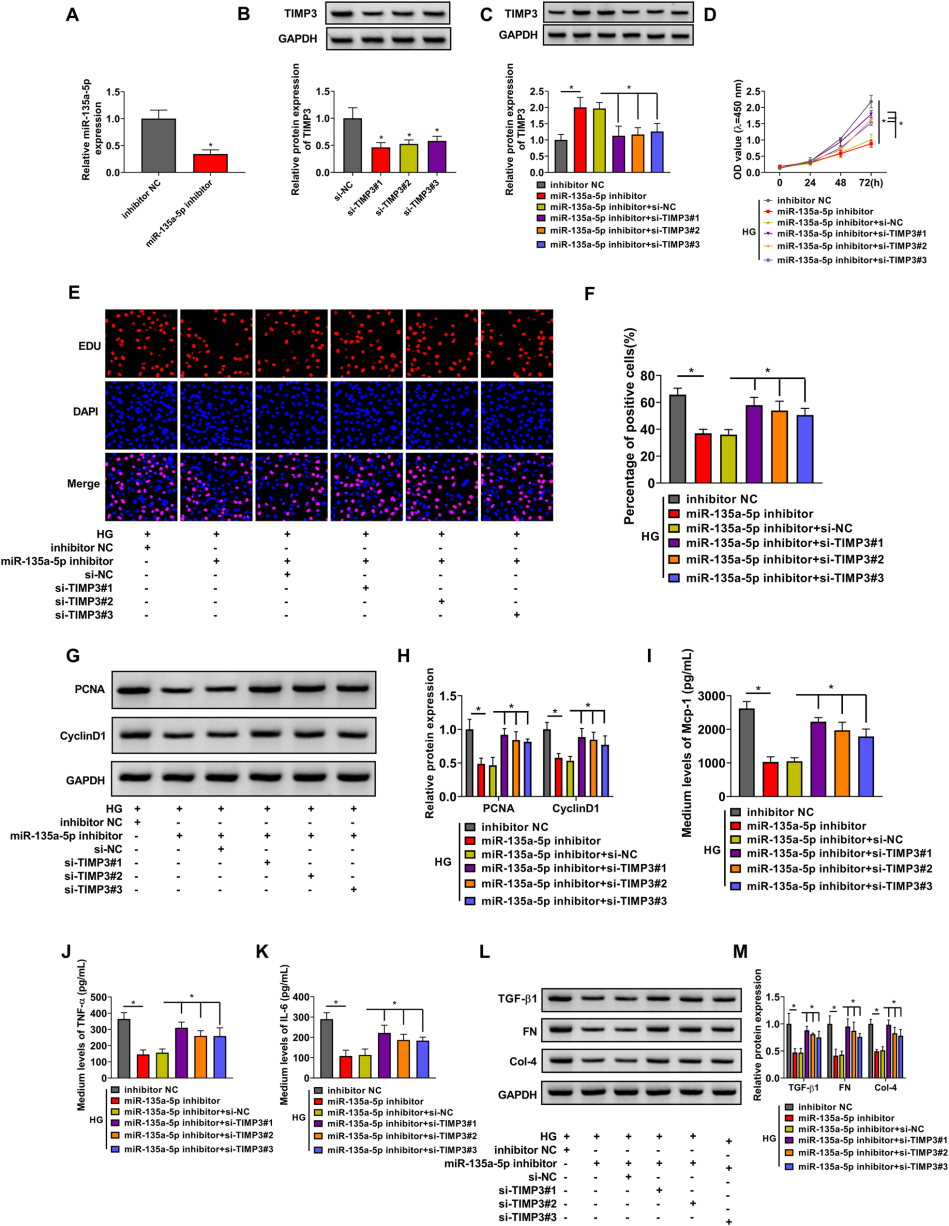
miR-135a-5p knockdown suppresses HG-induced effects in HMCs partly by up-regulating TIMP3. **A** The level of miR-135a-5p in HMCs transfected with inhibitor NC or miR-135a-5p inhibitor was determined by RT-qPCR. **B** The silencing efficiency of si-TIMP3 in HMCs was analyzed by Western blot assay. **C** HMCs were transfected with inhibitor NC, miR-135a-5p inhibitor, miR-135a-5p inhibitor + si-NC or miR-135a-5p inhibitor + si-TIMP3. Western blot assay was employed to assess the protein expression of TIMP3 in transfected HMCs. **D**–**M** HMCs transfected with inhibitor NC, miR-135a-5p inhibitor, miR-135a-5p inhibitor + si-NC or miR-135a-5p inhibitor + si-TIMP3 were induced by HG. **D**–**F** CCK8 assay and EDU assay were utilized to analyze cell proliferation ability. **G**, **H** Western blot assay was employed to detect the levels of PCNA and CyclinD1 in HMCs. **I**–**K** The levels of inflammatory cytokines (Mcp-1, TNF-α and IL-6) were analyzed by ELISA. **L**, **M** Western blot assay was utilized to analyze the expression of fibrosis-associated proteins in HMCs. *P < 0.05

We performed rescue experiments through transfecting HMCs with miR-135a-5p inhibitor alone or together with the siRNA of TIMP3 (si-TIMP3#1, si-TIMP3#2, or si-TIMP3#3). The silencing efficiency of miR-135a-5p inhibitor was high in HMCs (Fig. 5A). Also, Western blot assay confirmed the high interference efficiencies of si-TIMP3#1, si-TIMP3#2, and si-TIMP3#3 in HMCs (Fig. 5B). Transfection with miR-135a-5p inhibitor significantly up-regulated the protein expression of TIMP3, whereas the addition of TIMP3 siRNA reduced the expression of TIMP3 again (Fig. 5C). miR-135a-5p interference suppressed the proliferation in HG-treated HMCs (Fig. 5D, F). The results of Western blot assay also manifested that miR-135a-5p knockdown down-regulated the levels of PCNA and CyclinD1 in HG-treated HMCs (Fig. 5G, H). In addition, we found that the knockdown of miR-135a-5p restrained the inflammatory response (Fig. 5I, K) and fibrosis (Fig. 5 L, M) of HG-induced HMCs. With the interference of TIMP3, cell proliferation ability was largely recovered on the basis of the results of CCK8 assay, EDU assay and Western blot assay (Fig. 5D, H). TIMP3 knockdown also largely rescued the inflammation and fibrosis in miR-135a-5p-silenced HMCs upon HG treatment (Fig. 5I, M). These results indicated that miR-135a-5p knockdown attenuated HG-induced proliferation, inflammation and fibrosis of HMCs partly through enhancing the level of TIMP3.

### HG treatment increases the phosphorylation level of JNK partly by targeting CASC2/miR-135a-5p/TIMP3 axis

**Fig. 6:**
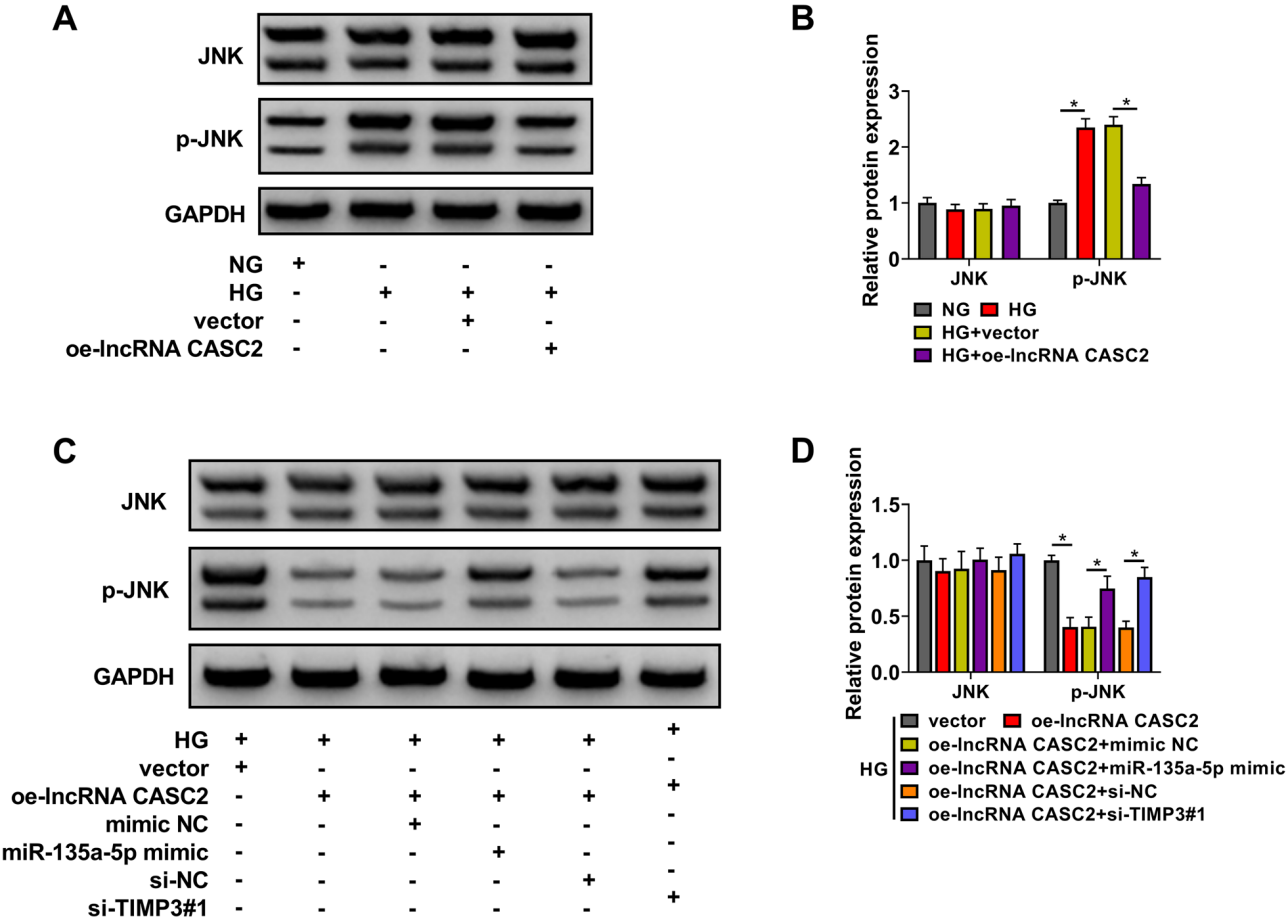
HG treatment increases the phosphorylation level of JNK partly through targeting CASC2/miR-135a-5p/TIMP3 axis. **A**, **B** The levels of JNK and p-JNK were detected in HMCs treated with NG, HG, HG + vector or HG + oe-lncRNA CASC2 by Western blot assay. **C**, **D** HMCs transfected with oe-lncRNA CASC2 alone or together with miR-135a-5p mimic or si-TIMP3 were stimulated by HG for 48 h. The levels of JNK and p-JNK were determined by Western blot assay. *P < 0.05

JNK signaling is identified to be aberrantly activated in DN patients, and suppressing the activation of JNK signaling is a novel treatment strategy for DN patients [24, 25]. HG treatment had no effect in the total protein level of JNK, but markedly increased the phosphorylation level of JNK in HMCs (Fig. 6A, B). The overexpression of CASC2 suppressed HG-induced phosphorylation of JNK (Fig. 6A, B). The overexpression of miR-135a-5p or the silence of TIMP3 partly rescued the phosphorylation level of JNK in CASC2-overexpressed HMCs upon HG treatment (Fig. 6C, D). Moreover, the results in Additional file 1: Figure S1 presented that the effect of CASC2 overexpression was slightly weaker than the effect of JNK1/2 inhibitor SP600125 in inactivating JNK signaling in HG-induced HMCs, suggesting that CASC2 plays a role similar to the JNK1/2 inhibitor SP600125 in HG-induced HMCs. These data demonstrated that CASC2 overexpression inactivated JNK signaling partly through targeting miR-135a-5p/TIMP3 axis in HG-induced HMCs.

## Discussion

Accumulating evidence have demonstrated that lncRNAs play important regulatory roles in the progression of DN [26, 27]. For instance, lncRNA XIST is reported to alleviate HG-induced damage in podocyte in DN through mediating miR-30/AVEN signaling cascade [28]. Ji et al. reported that the silence of lncRNA MIAT attenuates HG-induced proliferation and fibrosis in mesangial cells by down-regulating E2F3 level [29]. Li et al. found that lncRNA KCNQ1OT1 interference alleviates HG-induced proliferation, oxidative stress and deposition of ECM in HMCs through regulating miR-18b/HMGA2 signaling [7]. As for lncRNA CASC2, Min et al. found that CASC2 attenuates DN development by targeting miR-144/SOCS2 axis [9]. Zhang et al. found that CASC2 is down-regulated in the serum samples of DN patients and HG-treated HMCs, and CASC2 inhibits HG-mediated proliferation, oxidative stress and the accumulation of ECM in HMCs by regulating miR-133b/FOXP1 signaling [10]. We built DN cell model through exposing HMCs to HG. We found that HG time-dependently reduced the expression of CASC2 in HMCs. Furthermore, CASC2 overexpression alleviated HG-induced proliferation, inflammatory response and fibrosis of HMCs. These results suggested that CASC2 was a potential target for the intervention of DN progression.

Subsequently, we intended to investigate the molecular mechanism behind the biological function of CASC2 in DN. Accumulating studies have shown that lncRNAs exert their biological roles by acting as miRNA sponges [30, 31]. For example, lncRNA SNHG8 is reported to contribute to the development of prostate cancer by elevating HOXB7 expression via sponging miR-384 [32]. lncRNA MCM3AP-AS1 is reported to restrain the development of colorectal cancer through mediating miR-19a-3p/FOXF2 signaling [33]. We predicted the possible miRNA targets of CASC2 using LncBase database, and miR-135a-5p was confirmed as a target of CASC2 by dual-luciferase reporter assay and RIP assay. He et al. found that miR-135a is significantly up-regulated in DN patients, and miR-135a accelerates the renal fibrosis by targeting TRPC1 in DN [14]. Peng et al. demonstrated that circular RNA_010383 silencing facilitates DN progression by functioning as a sponge for miR-135a [34]. Zhang et al. claimed that miR-135a-5p level is enhanced in the serum and renal tissues of DN patients, and miR-135a-5p interference suppresses DN progression by regulating SIRT1 [35]. Consistent with these articles, we found that HG up-regulated the expression of miR-135a-5p in a time-dependent manner in HMCs. CASC2 overexpression-mediated protective effects in HG-induced HMCs were largely overturned by the addition of miR-135a-5p mimic, suggesting that CASC2 overexpression attenuated HG-induced effects in HMCs largely by down-regulating miR-135a-5p.

To further explore the working mechanism of miR-135a-5p in DN progression, the downstream targets of miR-135a-5p were predicted using StarBase database. TIMP3 was verified as a target of miR-135a-5p. TIMP3 is reported to exert important function in maintaining kidney homeostasis, and the down-regulation of TIMP3 is identified as a marker for DN [20]. Chen et al. found that miR-21 contributes to DN progression through reducing the expression of TIMP3 [36]. Wang et al. found that lncRNA TUG1 inhibits DN progression by sponging miR-21 to induce the expression of TIMP3 [37]. These results confirmed the protective role of TIMP3 in DN. We found that HG down-regulated TIMP3 level in a time-dependent manner in HMCs. CASC2 can positively regulate TIMP3 expression by acting as miR-135a-5p sponge in HMCs. Compensation experiments revealed that miR-135a-5p silencing attenuated HG-induced effects in HMCs partly by up-regulating TIMP3.

The abnormal activation of JNK signaling contributes to DN progression [24], and JNK signaling has been regarded as a therapeutic target for DN patients [25]. We found that HG treatment elevated the phosphorylation level of JNK in HMCs, and CASCs overexpression suppressed HG-induced phosphorylation of JNK partly through targeting miR-135a-5p/TIMP3 signaling.

However, there were several limitations of our study that should be noted. There were no clinical data about the expression of CASC2/miR-135a-5p/TIMP3 axis in DN patients and healthy volunteers, and only one cell line was chosen in this study. Furthermore, in vivo experiments were lacking in this study. Therefore, the results may be unpersuasive to some extent. In future, more study needs to be conducted to verify the results in this study.

**Fig. 7:**
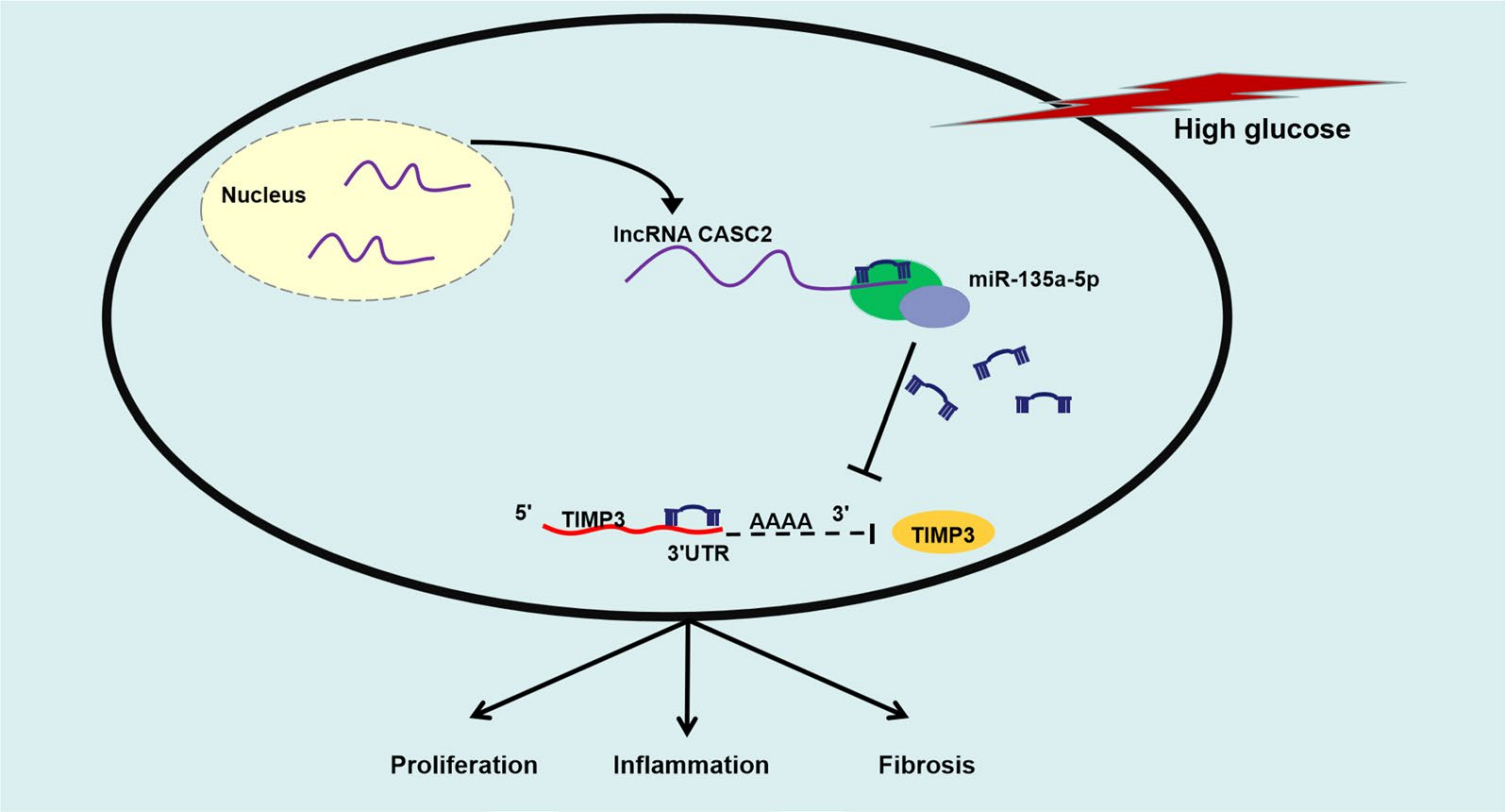
HG treatment promotes the proliferation, inflammation and fibrosis of HMCs partly through regulating CASC2/miR-135a-5p/TIMP3 signaling

In conclusion, HG treatment up-regulated the expression of miR-135a-5p and reduced the levels of CASC2 and TIMP3 in HMCs. CASC2 protected HMCs from HG-induced proliferation, inflammation and fibrosis largely through targeting miR-135a-5p/TIMP3 signaling (Fig. 7).

## Supplementary Information

**Additional file 1: Figure S1.** CASC2 plays a role similar to the JNK1/2 inhibitor SP600125 in HG-induced HMCs. The levels of JNK and p-JNK were detected in HMCs in the following five groups by Western blot assay: NG, HG, HG + vector, HG + oe-lncRNA CASC2, and HG + SP600125 (20 µM/24 h). *P < 0.05.

## Data Availability

Not applicable.

## Abbreviations

DN: Diabetic nephropathy
lncRNA: Long non-coding RNA
CASC2: Cancer susceptibility candidate 2
miR-135a-5p: microRNA-135a-5p
RT-qPCR: Real-time quantitative polymerase chain reaction
CCK8: Cell Counting Kit-8
ELISA: enzyme-linked immunosorbent assay
