# Supplementary figures and images for: Long non-coding RNA CASC2 restrains high glucose-induced proliferation, inflammation and fibrosis in human glomerular mesangial cells through mediating miR-135a-5p/TIMP3 axis and JNK signaling
Source: Diabetol Metab Syndr. 2021 Aug 26;13:89. doi: 10.1186/s13098-021-00709-5 (PMC8393478; doi:10.1186/s13098-021-00709-5)

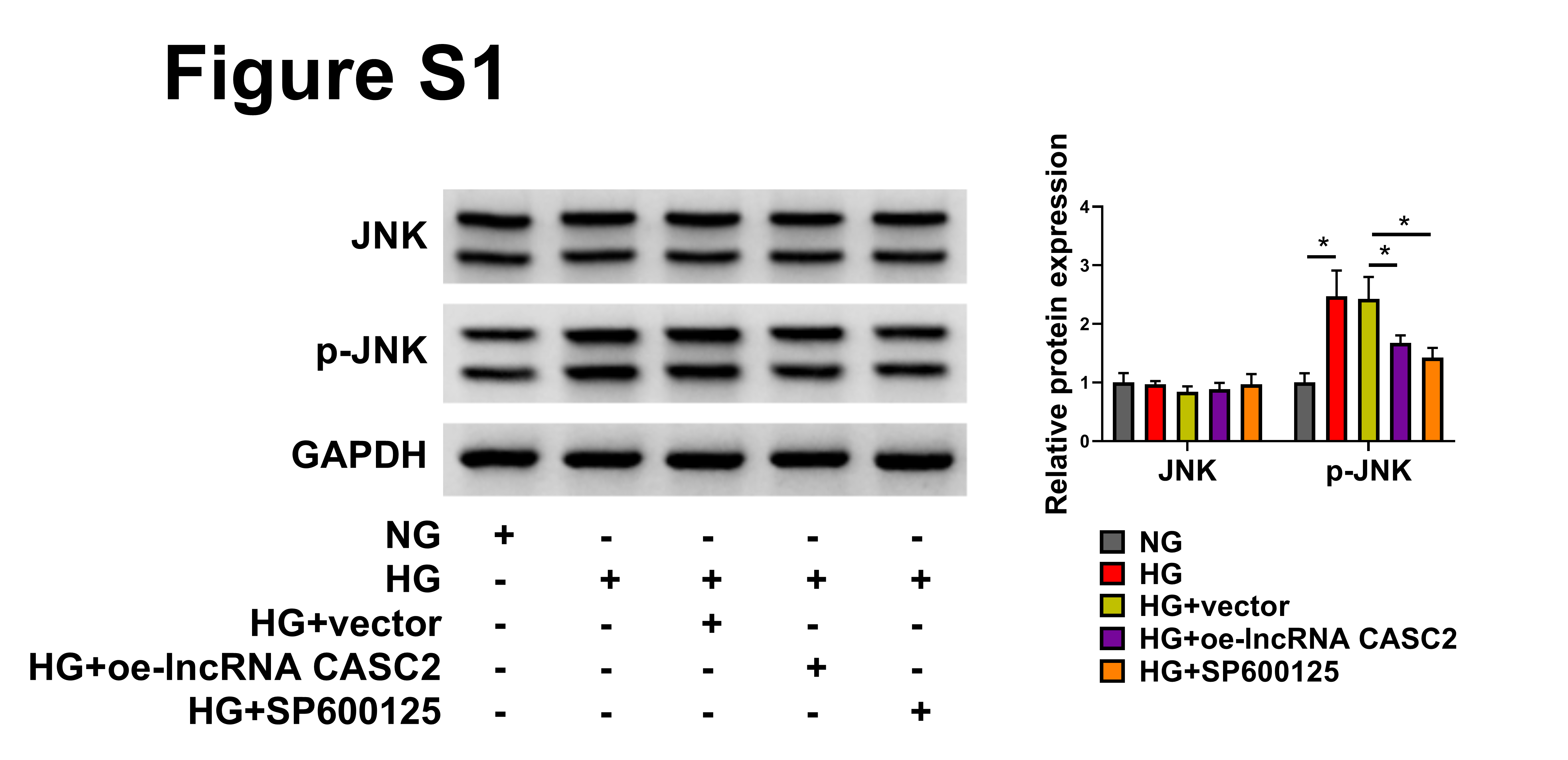

Supplement: Supplementary file 1 — Additional file 1: Figure S1. CASC2 plays a role similar to the JNK1/2 inhibitor SP600125 in HG-induced HMCs. The levels of JNK and p-JNK were detected in HMCs in the following five groups by Western blot assay: NG, HG, HG + vector, HG + oe-lncRNA CASC2, and HG + SP600125 (20 µM/24 h). *P < 0.05. [file 13098_2021_709_MOESM1_ESM.tif]
